# Nanoparticle Exposure and Workplace Measurements During Processes Related to 3D Printing of a Metal Object

**DOI:** 10.3389/fpubh.2020.608718

**Published:** 2020-11-25

**Authors:** Alexander C. Ø. Jensen, Henrik Harboe, Anders Brostrøm, Keld A. Jensen, Ana S. Fonseca

**Affiliations:** ^1^The National Research Centre for the Working Environment, Copenhagen, Denmark; ^2^Joblife A/S, Kolding, Denmark; ^3^Technical University of Denmark, DTU Nanolab – National Centre for Nano Fabrication and Characterization, Kgs Lyngby, Denmark

**Keywords:** additive manufacturing, selective laser melting, SLM, aerosol exposure, working environment, TiO_2_, ultrafine particles

## Abstract

Metal 3D printing has many potential uses within prototyping and manufacturing. Selective laser melting (SLM) is a process that uses metal powders in the micrometer range as printing material. The particle release from the entire SLM printing process is not well-studied. While the 3D printing itself often occurs in a sealed chamber, activities related to the process can potentially release harmful metal particles to the indoor working environment through resuspension of the printing powder or via incident nanoparticles generated during printing. The objective of this study was to improve the understanding of particle exposure in work processes associated with 3D printing and potential needs for interventions by a case study conducted in a 3D printing facility. In this setting, direct release and dispersion of particles throughout the workspace from processes related to metal 3D printing was investigated. The release from five activities were studied in detail. The activities included post-printing cleaning, object annealing, and preparation of new base substrate for the next printing was. Three of the five measured activities caused particles number concentrations in the working environment to increase above background levels which were found to be 8·10^2^ cm^−3^. Concentrations during chamber emptying and the open powder removal system (PRS) cleaning processes increased to 10^4^ and 5·10^3^ cm^−3^, respectively, whereas grinding activity increased number concentrations to 2.5·10^5^ cm^−3^. Size distributions showed that particles were mainly smaller than 200 nm. Respirable mass concentrations were 50.4 μg m^−3^, collected on filters. This was corroborated by respirable mass measured with a DustTrak of 58.4 μg m^−3^. Respirable mass concentrations were below the occupational exposure limits in Denmark for an 8 h time-weighted average.

## Introduction

Additive manufacturing (AM), also known as 3D printing, has increased in popularity during the last decade. One of its major advantages is rapid production prototyping, which reduce cost, redesigning the value chain in the early stage of product development (1, 2). AM is still in the early stage of implementation but has seen a 25% growth per year, while manufactured parts has increased by 80% in units (1). AM is a bottom up production technique that allows for more complex structures and has the potential of reducing material imperfections that would otherwise arise from casting or injection molding (1, 3). In AM, objects are produced by creating each layer sequentially from the bottom up, opposed to a traditional top down process where material is removed sequentially to produce the object. In general, AM is a combination of 18 manufacturing technologies that are either liquid-, solid-, or powder-based (4). In liquid-based AM, polymers are chemically activated and polymerized using lithography and direct light based techniques to form each layer. Solid based AM covers fused deposition, where heat is used to melt solid plastics or polymers and fuse it to subsequent layers. Powder-based AM covers metal and ceramics 3D printing, where Laser Powder Bed Fusion (L-PBF), which is the process used in selective laser melting (SLM) and direct metal laser sintering (DMLS), is the most used method (5). Here feedstock powders fusing in layers by selective illumination with a laser by either melting the powder completely for SLM or partially melting it and then sintering it in the case of DMLS. The powder is generally layered in thicknesses down to 20–50 μm using powder with a median grain size of 40–150 μm (6).

Laser-based metal 3D printing involve highly localized heat to fuse the feedstock metal powder. This process is similar to laser welding and may therefore release metal fumes similar to open welding processes. Open welding causes unintentional nanoparticle release (<100 nm) due to condensation of vaporized metal and spatter from the process. This leads to formation of metal oxides as well as ejected powder from the powder bed (6–13). Primary particles from welding are in the 10–150 nm range, which normally grow as function of times as a result of agglomeration and condensation processes (14). The L-PBF is usually a closed process under non-oxidizing atmosphere, N_2_ or Ar gas, to avoid generation of metal oxide nanoparticles. However, metal oxides and metal nanoparticles can be formed, similar to welding processes. Metal oxides and metal nanoparticles from welding are known to cause adverse health effects if inhaled (10, 15). Some of the most common metal constituents of the feedstock powders used in metal 3D printing are titanium, iron, vanadium, and aluminum. Titanium may form TiO_2_ in the presence of oxygen. Nano-size TiO_2_ particles has been shown to cause genetic damage in the respiratory system *in vivo* and are therefore classified by the EU as potentially carcinogenic (16). Iron and iron oxide particles can cause fibrotic and non-fibrotic damage in the lungs and potentially lead to pneumoconiosis (17). Vanadium particles are involved in acute respiratory diseases and possible neurotoxic (18, 19). Aluminum causes neurotoxicological effects and pulmonary fibrosis (20, 21). Any release of such respirable, i.e., PM_4_, metal or metal oxide particles from powders or unintentional particles can thus be a potential hazard to the workers. The predominant route of exposure for workers who are in contact with respirable particles is the inhalation pathway, however dermal and oral exposure pathways should not be disregarded (22). Often in metal 3D printing, the feedstock powder is reused several times, which may lead to changes in particle size distribution and give rise to unexpected exposure and effects on human health, safety, and environmental burden. Du Preez et al. (23) conducted material characterization of metal Ti powders. They concluded that virgin and reused powder showed identical morphology and size distributions meaning that release will most likely not be dependent on initial state of the powder. However, Mellin et al. (24) found that smaller particles in the 1–5 μm range were formed as by-product of the printing process in the reused material when studying Nickel feedstock material. In both cases, particles in the respirable range were found in all electron microscopy (EM) samples.

There is limited information on the occupational exposure to feedstock powder and incidental particles released from metal 3D printing (24). Ljunggren et al. (25) concluded in their studies that the exposure is low but transient emissions might pose a concern. Ladewig et al. (26) found that by-products from the SLM printing process were similar to by-products formed during welding. Sousa et al. (27) found that post-processing activities have a potential to release incidental nanoparticles, while Graff et al. (6) found particle release in the size range from 10 nm to 10 μm from activities that were part of post processing of the printed object. An increase in particle number concentrations up to 1.6 × 10^4^ cm^−3^ were registered, but emissions based on the types of activities were not reported.

In the present study, we investigated the release and potential exposure to particles from the production chain related to 3D metal printing processes. Measured activities involved the cleaning and post-processing of an object printed using the SLM printing technique. The object was printed using a titanium-based metal powder with elemental composition Ti_6_Al_4_V. Total particle number concentrations and size distributions were measured in near field and far field positions using state-of-the-art aerosol instruments. Respirable mass in the breathing zone of the worker was collected and compared with occupational exposure limits.

## Methodology

### Work Environment and Measurement Locations

**Figure 1:**
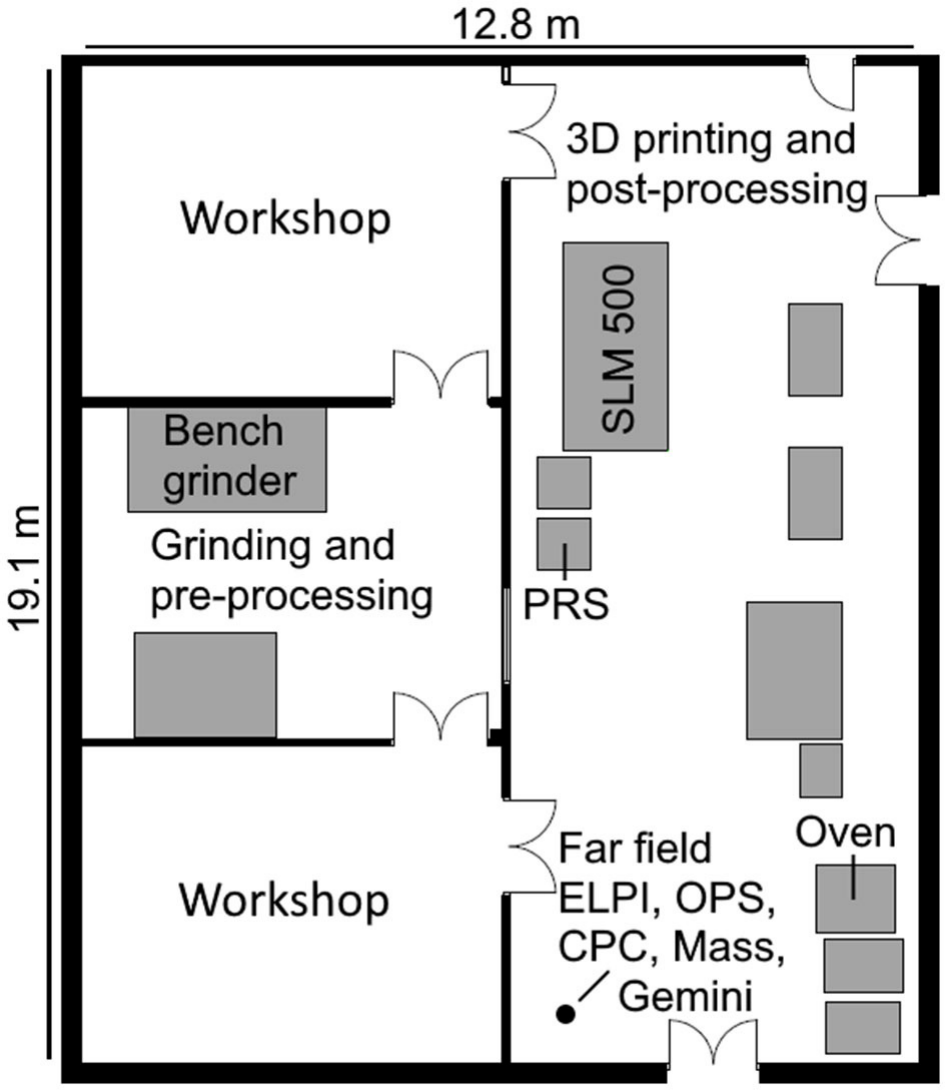
Layout of the working environment. Locations of machines used during processes as well as the stationary FF measurement position are marked. The figure was made to scale according to blueprints of the facility.

The measurements were conducted at Danish Technological Institute located in Aarhus, Denmark on the 17th of January, 2020 during the cleaning activity of the printing chamber and post-processing of a 3D printed metal object. The facility consisted of two rooms, one for pre-processing and printing with dimensions of 6.5 m × 6.5 m (l × w) and another for printing and post-processing of new substrates with dimensions 19.1 m × 6.5 m (l × w) (Figure 1). In the workplace, several workstations were used in sequence, based on the process in the pipeline for preparation, 3D printing, and post-processing of a metal object. The facility was ventilated at an unknown flow rate using mechanical forced ventilation system. Doors between areas were kept open at all times allowing air to mix between rooms. No other processes occurred in the workspace during measurements.

Real-time particle monitoring combined with collection of samples for gravimetric and morphological analysis during working and non-working periods were carried out simultaneously in the near field (NF), far field (FF), and breathing zone (BZ). The BZ was measured with personal measurement instruments that were put in a carry bag, worn by the worker with sampling tubes to the instruments mounted in the BZ. Since the processes occurred in various positions in the facility, the NF measurement position was moved according to the activity so that the inlets to the samplers were consistently at a height of 1.5 m and 125–130 cm from the activity (Supplementary Figure 1A). The NF was therefore considered as the volume around the process activity occupied by the worker during the studied process. The FF measurement position was constant throughout the measurement campaign, in the corner of the workspace, as indicated in Figure 1 and Supplementary Figure 1B. This meant that the distance from the FF to the NF location varied from 9 to 13 m. Humidity and temperature was monitored in the FF using Gemini TinyTagPlus (TGP-1500, Gemini Data Loggers Ltd., West Sussex, UK).

### Processes and Materials

The process emissions that were characterized include emissions from vacuum cleaning the 3D printing chamber and object, baking the 3D object, and preparing a new metal substrate for 3D printing by grinding the surface. These processes were conducted after printing the 3D metal object from Ti_6_Al_4_V feedstock powder (Tekna advanced materials Inc., Quebec, Canada) using a SLM metal 3D printer (SLM500, SLM Solutions, Lübeck, Germany).

The cleaning process was done using an external vacuum cleaner rated for M-risk material, i.e., fine dust, with a high volume flow rate to remove excess metal powder from the 3D printing chamber (Supplementary Figure 1C). The printed object was transferred to a table next to the SLM500. The 3D printing chamber itself is moved to a glovebox, powder removal system (PRS) fitted with an industrial vacuum cleaner certified for fine dust in class M, for further vacuum cleaning. The excess metal powder is led back into the powder reservoir for reuse. The rough cleaning was done in a closed system state. After the initial rough cleaning, the PRS was opened and the SLM printing chamber was cleaned carefully. The printed object is removed from the SLM printing chamber (Supplementary Figure 1D), transferred to an oven, and gradually heated from room temperature to 550°C overnight in nitrogen or argon atmosphere for annealing. Finally, a new substrate was prepared by grinding the surface of a piece of stainless steel (316L) using a flatbed table grinder (Supplementary Figure 1E).

The mass-based respirable dustiness of the Ti_6_Al_4_V feedstock powder, i.e., a materials' tendency to generate dust during processes which involve pouring and agitation of bulk powder, was determined by using the small rotating drum method (28, 29).

### Particle Monitoring and Sampling Techniques

The measurement strategy adopted in this study followed the harmonized 3-tier approach for particle exposure assessment published by the Organization for Economic Co-operation and Development (30). In this campaign, a combined approach of temporal and spatial analysis were adopted for discriminating background and process generated particles (31). The non-working periods were used as temporal approach to define the background (BG) concentrations at all measurement points using the measurements obtained prior to activity in the facility. The spatial analysis assumed the measurement location in FF being representative as BG concentrations.

The particle concentrations in the BZ of the worker were sampled using a portable instrument. A diffusion size classifier miniature (DiscMini, Matter Aerosol AG, Wohlen, Switzerland) was used to measure total particle number, mean particle diameter, and the lung deposited surface area (LDSA) of particles in the size range of 10–700 nm with 1 s time resolution. For the DiscMini, the particles were sampled through transparent conductive Tygon™ tubing (32), while electrically conductive silicone sampling lines were used for the rest of the instruments.

In both NF and FF measurement positions, optical particle sizers (OPS; TSI model 3330, TSI Inc., Shoreview, MN, USA) were used to measure the optical particle size distributions in 16 channels from 0.3 to 10 μm in 1 s intervals.

Additionally, portable condensation particle counters (CPC; TSI model 3007, TSI Inc., Shoreview, MN, USA) were used to measure the total number concentration of particles from 10 nm to >1 μm in 1 s time resolution.

Furthermore, in the NF a NanoScan SMPS (NanoScan; TSI model 3091, TSI Inc., Shoreview, MN, USA) was used to measure the particle mobility size distributions in 13 channels for sizes between 10 and 420 nm in 60 s intervals. A DustTrak (DustTrak DRX aerosol monitor 8533, TSI Inc., Shoreview, MN, USA) was used to measure the mass fractions of the aerosol divided into total mass, PM_10_, respirable, PM_2.5_, and PM_1_ in 60 s intervals. Respirable mass was collected using a filter sampler with a cyclone pre-impactor GK2.69 (BGI Inc., Waltham, MA, USA) or SCC1.062 (BGI Inc., Waltham, MA, USA), sampling at 4.2 or 1.05 L min^−1^, respectively.

In the FF, aerodynamic particle size distributions were additionally measured using an electrical low-pressure impactor (ELPI+, Dekati, Finland) which measures particle size distribution and number concentration. The ELPI was used in high-resolution mode, which results in 100 channels between 6 nm and 10 μm with 1 s time resolution. In the FF, respirable mass (PM_4_) was sampled using a SCC1.062 (BGI Inc., Waltham, MA, USA) cyclone, sampling at 1.05 L min^−1^.

The respirable dust was collected for gravimetric and inorganic chemical analysis on Fluoropore™ membrane filters 37-mm PTFE with 0.8-μm pore size (Millipore, Billerica, MA, USA) mounted in sampling cyclones connected to portable sampling pumps (Apex2, Casella Inc.). Particle mass concentrations were gravimetrically determined by pre- and post-weighing the filters collected using an electronic microbalance (Mettler Toledo Model XP6) with ±1 μg sensitivity located in a climate controlled weighing room (RH = 50%, T = 22°C). The filters were acclimatized for a minimum of 24 h at 50% relative humidity before weighing. Three blind filters were stored and used as laboratory blanks to correct for handling and environmental factors.

Three different samples were collected for analysis by scanning electron microscopy coupled with energy dispersive x-ray spectroscopy (SEM/EDS). The samples were collected on 400-mesh Cu grids pre-coated with holey carbon film with a mini-particle sampler (MPS) (33) connected to a pump (Apex2, Casella Inc.) operating at 0.3 L min^−1^. The first sample was collected during the open PRS process (referred to as PRS-O), while the two others were collected during grinding activity (referred to as Grind1 and Grind2). Sparks were only observed from the grinding process when collecting the Grind2 sample. The samples were analyzed in high vacuum mode with a NOVA NanoSEM (Thermo Fisher Scientific, The Netherlands) situated at the Technical University of Denmark (DTU). Samples were analyzed at either 10 or 20 keV, using an aperture size of 50 μm and a spot number of 4.5 with probe currents of 0.16 and 0.32 nA, respectively. An XFlash FlatQuad (Bruker Nano, Germany) EDS detector was used to measure elemental composition of the samples via mapping. The detector is annular and situated directly above the sample, resulting in a solid angle close to 1 sr. All EDS maps were acquired with a 128 μs dwell time, resulting in an acquisition time of 15 min per map. They were analyzed with the Feature analysis and Mapping functions of the ESPRIT 2 software (Bruker Nano, Germany). Segmentation was performed on images generated from EDS signals rather than the SE signal to avoid misclassification of the holey substrate and from dried residues on the sample. To capture all relevant particle types, the segmentation was performed on images generated from the Al-, Si-, Cl-, Ti-, and in some cases also the Ca-signals. These elements were chosen as they represent each of the different particle types, identified when inspecting the SE images overlaid by their corresponding EDS map. Once the segmentation was performed, the x-ray spectra from pixels within each recognized particle were summed and quantified using the Cliff-Lorimer model as recommended by Brostrøm et al. (34). Particles were classified based on their elemental composition, using the classification scheme presented in Table 1. Particles were filled into classes starting from the top of Table 1, meaning that once the criteria of a class were fulfilled, the given particle was assigned to it and the classification moved on to the next particle. Thus, some particles could have fulfilled the criteria of several classes, but were only assigned to the first hit, which may have resulted in an artificially high number of the upper classes.

**Table 1:** Classification scheme based on the atomic percentage (at%) used to distinguish particle types based on their elemental composition.

**Classes**	**Ti [at%]**	**Cl [at%]**	**Si [at%]**	**Na [at%]**	**Ca [at%]**	**Al [at%]**	**C [at%]**
Ti-based	≥5	–	–	–	–	–	–
Si-based	–	–	≥5	–	–	–	–
NaCl	–	≥5	–	≥5	–	–	–
Ca-based	–	–	–	–	≥5	–	–
Al-based	–	–	–	–	–	≥25	–
C-based	–	–	–	–	–	–	≥70
Unclassified	<5	<5	<5	<5	<5	<25	<70

To avoid misclassification, limits were set at an atom percentage (at%) of 5, except for Al and C, which ensured that particles falling into a given class contained a significant amount of the relevant element. This was necessary since the recorded x-ray spectra from nano-sized particles can have very low counts, resulting in high uncertainties when quantifying for trace elements with at% approaching 1%. Higher limits were needed for C and Al, as these elements had a higher background signal, originating from the holey carbon substrate and the samples holder, respectively.

The maps were analyzed using ESPRIT 2 (Bruker Nano, Germany). In addition to EDS analysis, all samples were imaged with an Everhart Thornley secondary electron detector, where images were acquired at magnifications of 5–20 k, corresponding to pixel resolutions between 14.6 and 7.6 nm/pixel.

### Data Processing

All online instruments were time-synchronized and inter-compared before the activity in the facility. Particle concentrations have been corrected for diffusion losses in sampling tubes where possible according to Cheng (35). The cumulative workers exposure for an 8-hour time-weighted average (8 h-TWA) was estimated according to Equation (1).

(1) $$\begin{array}{r} 8hTWA=\;\frac{t_{1}C_{1}+t_{2}C_{2}+\ldots +t_{n}C_{n}}{t_{1}+t_{2}+\ldots +t_{n}} \end{array}$$

where *C*_*n*_ is the measured particle concentrations subtracted by the background concentrations during a specific operation and *t*_*n*_ is the time duration of the activity.

In this study, an equivalent workers exposure of a typical working shift during post-processing of 3D metal printing was considered of 2 h total daily duration. For the remainder of the 8 h working shift the FF concentrations were used as representative for the exposure assessment.

## Results

Respirable dustiness mass-fraction obtained for the Ti_6_Al_4_V feedstock powder used for the 3D printing was found to be 171 ± 5.1 mg kg^−1^). According to the EN 15051 ranking system (36), the applied feed-stock material falls within the category of powders with moderate respirable fraction dust mass release (between 50 and 250 mg kg^−1^).

Average temperature and humidity in the workplace during the activities was measured to 21.2°C and 34.8% RH, respectively, which is slightly lower % RH than applied in dustiness testing (50% RH).

**Figure 2:**
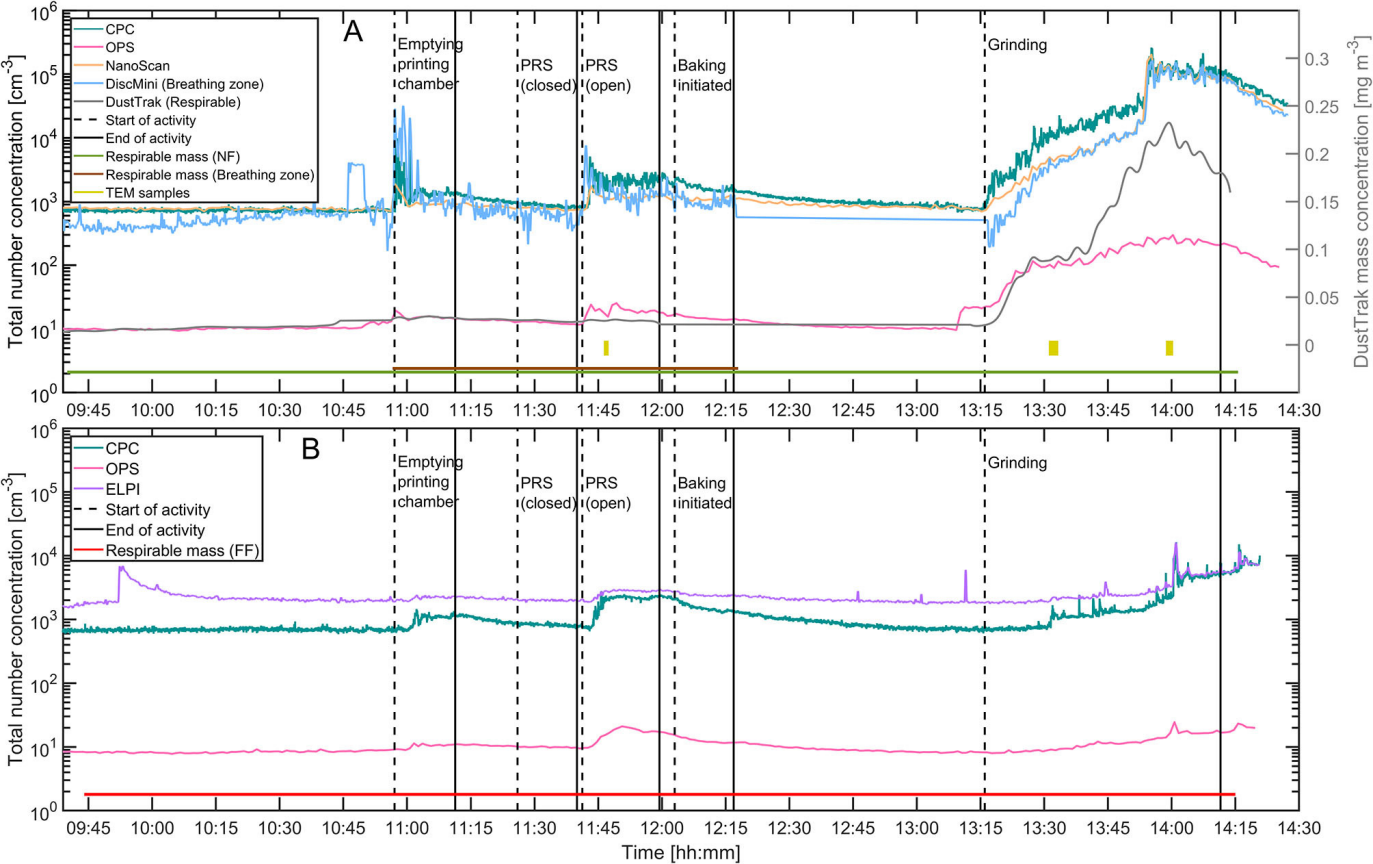
Total number concentrations and respirable mass measured in the NF and BZ **(A)** and FF **(B)**. Start and end of activities are indicated with dashed and full lines, respectively. Times where respirable mass of filter samples and SEM samples were collected are indicated with horizontal lines at the bottom.

Total particle number concentrations measured in the BZ, NF, and FF are shown in Figures 2A,B, respectively. Respirable mass concentrations measured by the DustTrak in the NF is shown in Figure 2A. The ELPI, DiscMini, and DustTrak concentration data in Figure 2 were smoothed using a 10 s running average to reduce the influence of electrical noise from the electrometers and improve overall readability.

Prior to the start of the first process, the background concentrations were measured. For both BZ, NF, and FF the background concentrations were 8·10^2^ cm^−3^ (Figure 2). Three out of five measured activities caused an increase in terms of particle number concentrations in the working environment whereas the remaining two processes did not affect particle concentrations in the measured size range. The duration of each of the activities is found in Table 2.

**Table 2:** Activity duration, mean number concentration, N, particle size, D_p_, and LDSA measured by the DiscMini in the BZ.

**Activity**	**Activity duration [min]**	**N [10^**3**^ cm^**−3**^]**	**D_**p**_ [nm]**	**LDSA [μm^**2**^ cm^**−3**^]**
Emptying printing chamber	15	4.2	58	7.6
PRS closed	14	0.6	67	2.9
PRS open	18	1.5	60	4.6
Baking initiated	14	1.0	70	3.8
Grinding	55	36.1	53	79.3

### Emptying Printing Chamber and Powder Removal System

**Figure 3:**
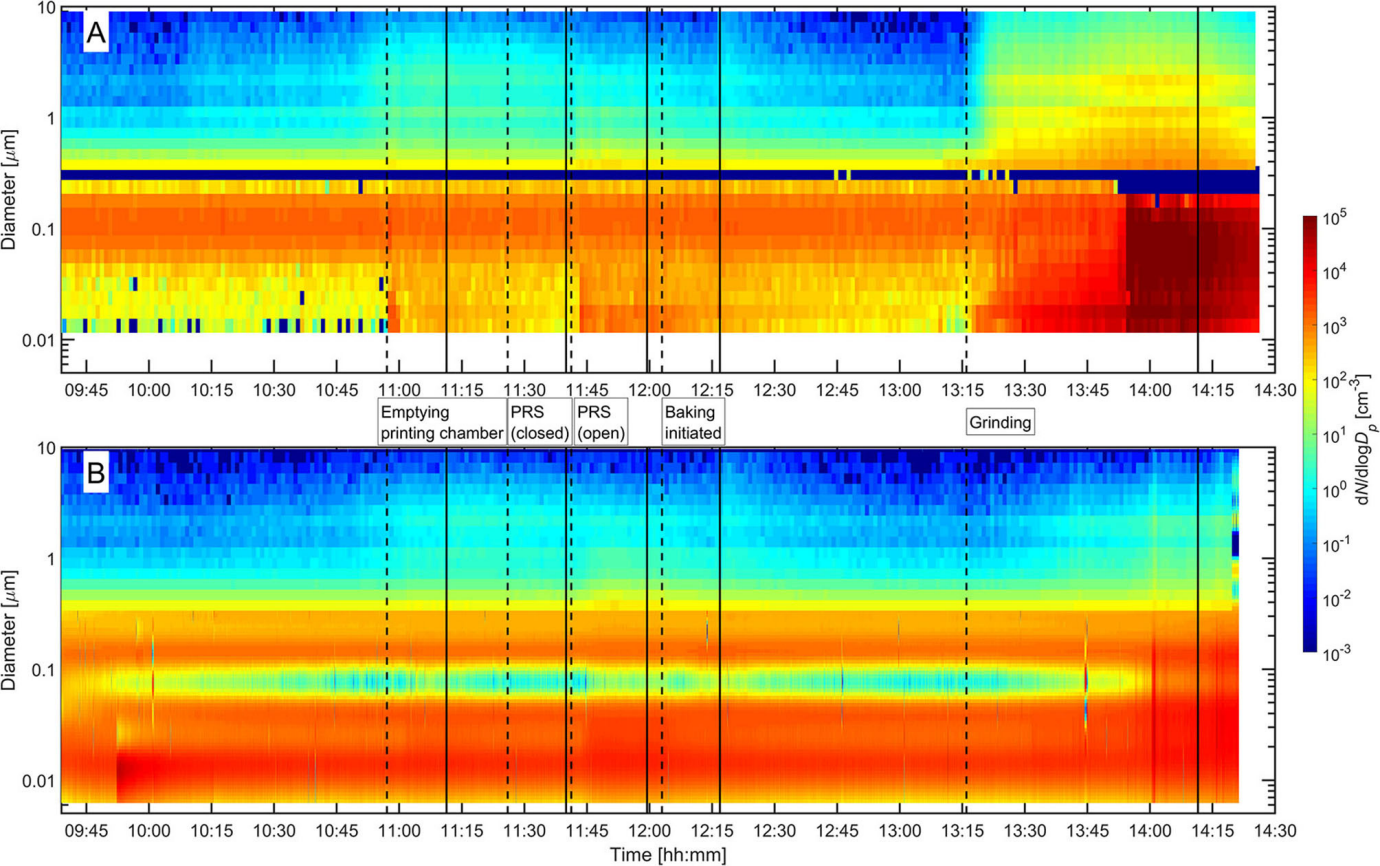
Particle size distributions measured with the NanoScan and OPS in NF **(A)** and ELPI and OPS in the FF **(B)**. Start and end of activities are indicated with dashed and full lines, respectively.

Emptying of the printing chamber and removing the powder using the open PRS system showed an initial increase in total particle number concentration at the NF. These concentrations increased from background levels to 10^4^ and 5·10^3^ cm^−3^, respectively, for emptying and powder removal as measured by the CPC. After the initial increase, number concentrations decreased toward background concentrations as the activity progresses. Similar concentrations were measured with the DiscMini in the BZ meaning that mainly particles below 300 nm are released from the activity. Mean particle sizes and LDSA show that particles were between 53 and 70 nm in the BZ and LDSA were in the order of 3–7 μm^2^ cm^−3^ except for the grinding activity, which released one order of magnitude higher LDSA at 80 μm^2^ cm^−3^ (Table 2). This is supported by the particle size distributions measured by the NanoScan, which shows a mode around 100 nm (Figure 3).

In the FF, the particles reached 1.5·10^3^ and 2.5·10^3^ cm^−3^ measured by the CPC during emptying and the open PRS cleaning, respectively. The increase in particle number concentrations from work activity was delayed when comparing the NF and FF time series. In addition, FF concentrations were lower due to the distance between the activity and the FF location, which allowed mixing and dispersion in the indoor environment.

**Figure 4:**
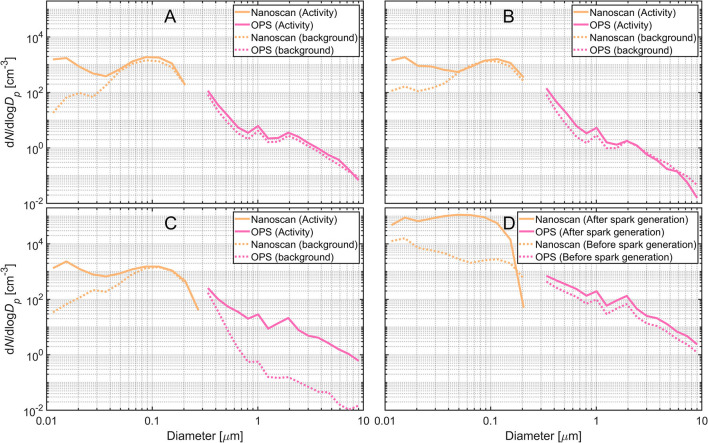
Particle size distributions during: **(A)** emptying the 3D printing chamber, **(B)** open PRS, **(C)** start of grinding, and **(D)** before and after the spark generation during grinding.

The particle size distributions show that particle modes related to the activity increased 10–100-fold in the NF (Figures 3A, 4). When emptying the 3D printing chamber, particles with a mode of 15 nm were dominant (Figure 4A). For the open PRS removal activity, a much broader mode of particles around 28 nm was detected in addition to the 15 nm mode (Figure 4B).

### Grinding a New Substrate

For the grinding activity, particle concentrations increased during the entire process, which indicates a high release rate to the working environment. Particles were mostly metallic and formed due to sparks generated during the abrasion as well as droplets consisting of a water and soap mixtures from the cooling system. Particle concentrations during grinding increased up to a maximum of 2.5·10^5^ and 1.5·10^4^ cm^−3^ in the NF and FF, respectively.

Particles released at the start of the grinding activity caused increase in particle concentrations for particles in sizes of 15 nm and particles above 500 nm to increase in. Sparks from the grinding process caused an additional broad mode in the range 50–80 nm (Figures 4C,D).

The respirable mass concentrations collected on filters in the BZ, NF, and FF showed that the mass concentrations in the BZ and FF were below detection limit (DL) for the filters (<3.9 μg) (Table 3). The NF respirable mass concentration was 50.4 μg m^−3^. The respirable mass concentration measured by the DustTrak was 58.4 μg m^−3^. The DustTrak mass concentration was calculated as the average concentration during the same period as the NF respirable mass was sampled.

**Table 3:** Respirable filter concentrations and mean near field respirable mass concentrations measured by the DustTrak for the same period as NF respirable mass was collected.

**Location**	**Concentration [μg m^**−3**^]**
Personal breathing zone	< DL
Near field	50.4
Far field	< DL
DustTrak (NF)	58.4

*DL, detection limit*.

### SEM/EDS Analysis

**Figure 5:**
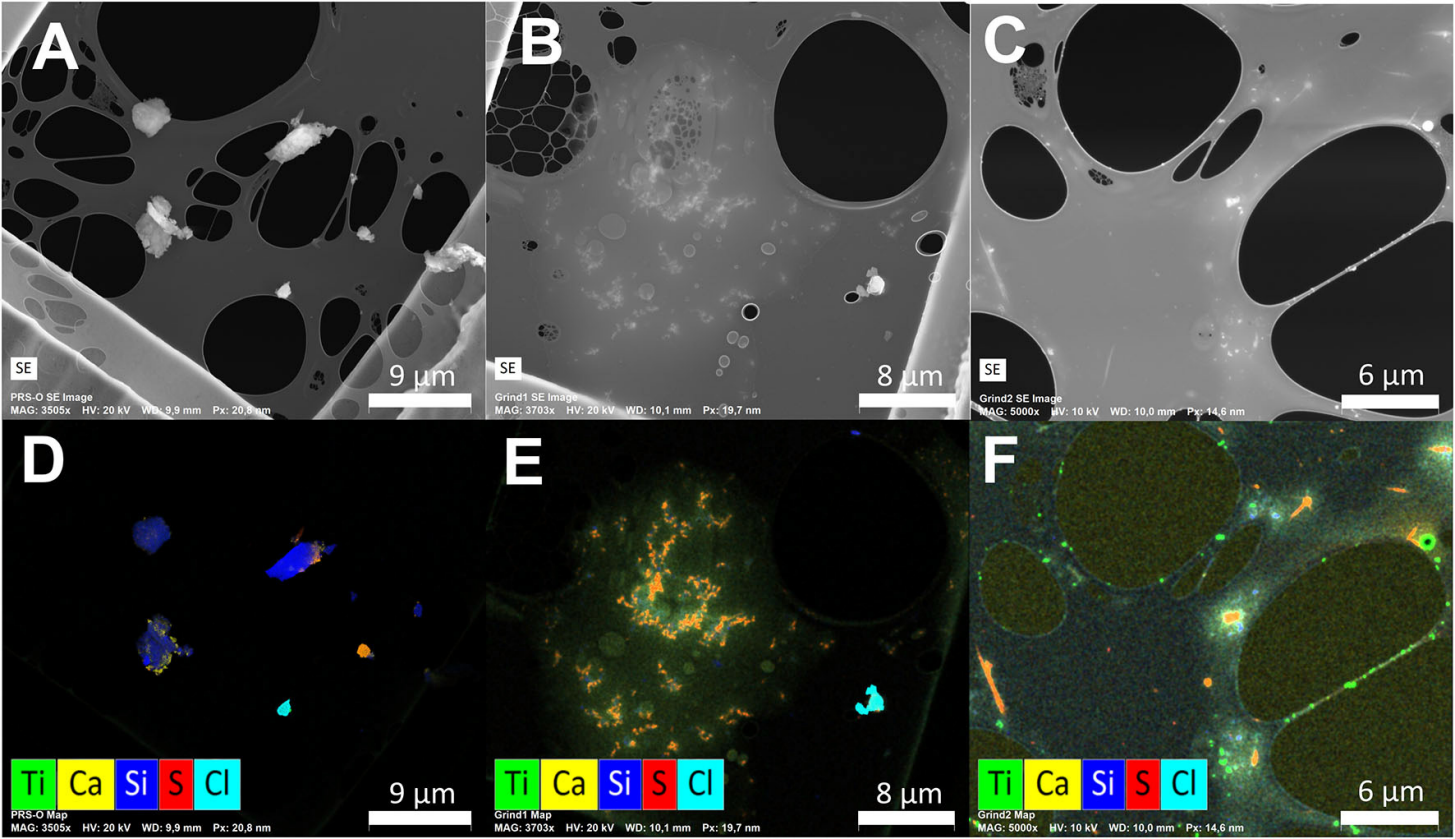
Representative SE images **(A–C)** and overlaying EDS maps of select elements **(D–F)** from the PRS-O sample **(A,D)**, Grind1 sample **(B,E)**, and Grind2 sample **(C,F)**. The EDS maps were generated based on X-ray counts in the energy range corresponding to an emission from the given element. The individual element maps were those used for segmentation prior to quantification.

An overview of results from the SEM/EDS analysis is provided in Table 4. It should be noted that the two Grind samples also contained large areas of dried droplets (Figure 5B), which have not been included in the number of detected particles. These droplets would have increased the total number of μm-sized particles, and potentially reduced the number of nm-sized particles, which were originally in the droplets. Examples of SE images and overlaying X-ray count EDS maps of select elements from each of the three samples are presented in Figure 5.

**Table 4:** Overview of selected settings and results from the SEM/EDS analysis.

**Sample**	**EDS maps, #**	**Resolution range [nm px^**−1**^]**	**Analyzed area [μm^**2**^]**	**Percentage of grid analyzed [%]**	**Particles detected [#]**	**Particle number density [μm^**−2**^]**
PRS-O	5	8.9–20.8	5,005	0.07	37	0.0074
Grind1	5	14.6–21.5	5,564	0.08	21	0.0038
Grind2	5	9.4–14.6	3,399	0.05	354	0.1041

**Figure 6:**
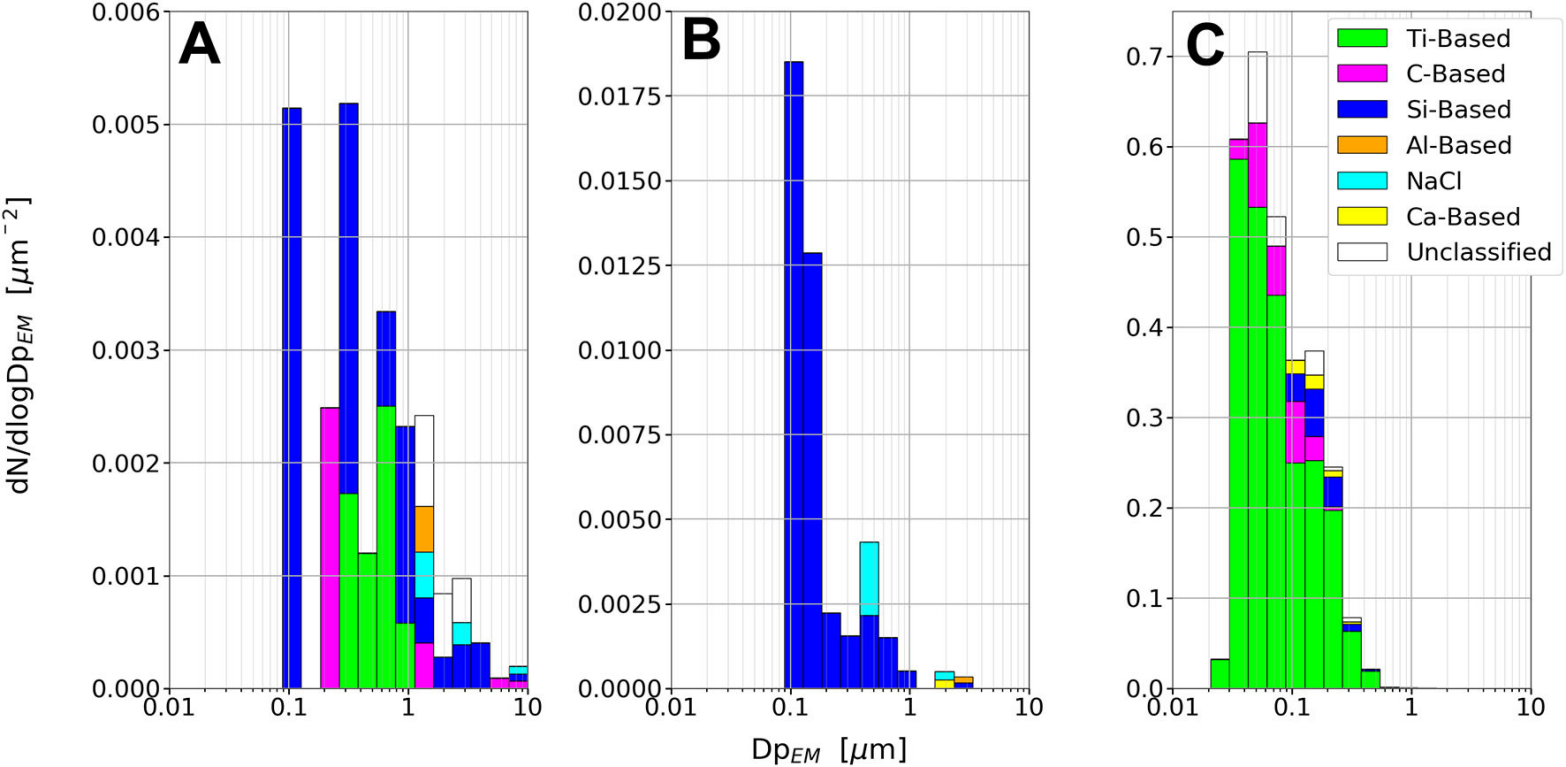
Class differentiated particle size distributions from the PRS-O sample **(A)**, Grind1 sample **(B)**, and Grind2 sample **(C)**. Note that the y-axis is scaled differently in each plots.

The PRS-O sample mainly consisted of environmental particles in the upper nm and μm range. The most common particle type was Si-based, consistent with crust and mineral particles from the outdoor environment. Various salt particle combinations were also observed with the main components: Ca, S, Cl, and Na, most likely in the form of NaCl, CaSO_4_, and CaCO_3_. Examples of Si-based, NaCl, and CaSO_4_ particles are presented in Figures 5A,D. In addition, some Ti-based particles were observed on the sample with diameters ranging from 0.2 to 1 μm (Figure 6A).

Most of the grid squares on the Grind1 sample contained areas with residues as those seen in Figures 5B,E. Within these areas a wide variety of elements were detected, including Ca, S, Cl, and to a minor extent Mg, K, Na, and Si, where Si was typically present as small particles embedded in the residue. The areas presumably resulted from droplets that were collected onto the TEM grid, and which dried in the vacuum of the microscope, leaving behind a variety of ions and low volatility compounds. Since the residue areas were not observed on the PRS-O sample, they were most likely generated as a result of the grinding process, which is consistent with the coolant added to the grinding process. The residue areas complicated the segmentation processes, as they made both the SE and EDS signals difficult to separate into a particle and substrate phase (Figures 5B,E). However, when using images generated from EDS signals of the individual elements it was still possible to distinguish particles consisting of elements other than Ca, S, and Cl. It should thus be possible to detect both Si- and Ti-based particles. However, no Ti-based particles were observed within the analyzed areas, whereas Si-based particles were still observed, indicating a low Ti particle concentration. The Si-based particles were primarily in the 100–200 nm size range (Figure 6B).

Unlike the two previous samples, the Grind2 sample consisted almost exclusively of Ti-based particles (Figures 5C,F). The Ti-based particles were in the size range 20–300 nm, with the main mode at 35 nm (Figure 6C). The sample was collected during the same process as the Grind1 sample, except that sparks were generated during sampling. This indicates that sparks during grinding correlate with the release of nano-sized Ti particles to the air.

## Discussion

Since the 3D printing process itself was contained in a closed chamber with efficient ventilation, the release from the 3D printing was not a main cause of concern for potential workers exposure and was therefore not measured. However, remains of metal powder used for printing or particles formed during the 3D printing process may re-suspend during handling and thus potentially lead to exposure of metal particles.

The respirable mass concentration in the BZ was only measured during emptying and cleaning activities and not during the grinding activity, where measured mass and total particle number concentrations were the highest (Figure 2A). This caused the mass on the filters to be below DL (<3.9 μg). Similarly, the respirable mass collected on filters in the FF was below DL due to low mass concentrations for the duration of the sampling period. Mass concentrations collected in the NF were consistent with DustTrak measurements.

Direct comparison between concentrations measured with particle counters using different methods of detection as well as with offline sampling should be done with care. As demonstrated by e.g., Fonseca et al. (37), Spinazzè et al. (38), and Borghi et al. (39) differences between readings of instruments can be significant.

Using the PRS system in a closed state, no particles were released from the process showing the value of a closed environment with efficient ventilation to limit release from the activity, as there was no significant leak of particles from the system to the surrounding working environment. With PRS chamber open the metallic powder was resuspended and released into the working environment dispersing to the NF and further to the remainder of the working volume as measured in the FF location.

By analyzing the size distributions at various time points during the grinding process it is possible to estimate the source of particles. The grinding process can be split into three steps. In the first step, the cooling solution was sprayed on the surface of the substrate. In the second step, a local exhaust ventilation was turned on behind the grinding wheel, opposite of the spray nozzle of the cooling liquid. In the third step, the grinding wheel reached the substrate, initiating the grinding and spark generation. At 13:30 the local exhaust ventilation was turned on at the bench grinder, which shows as a change in slope of the concentrations measured in the NF and BZ (Figure 2A). Particles with diameters larger than 500 nm during the grinding activity are mostly droplets generated from the cooling solution (Figures 4C,D). The smallest mode at 15 nm most likely consists of primary metal powder particles resuspended from the coolant solution. At 13:50 the grinding wheel reached the substrate and generated sparks (Figure 2 and Supplementary Figure 1E). This leads to a wider mode around 50–80 nm (Figure 4D). Before the grinding wheel reached, the substrate there is no significant mode at 50–80 nm compared with the background size distribution. The composition of the particles in the 50–80 nm mode as identified with the EDS revealed that they were mainly Ti-based, which is consistent with the printed material on the base plate that were ground away to prepare the base plate for the next printing. Particles measured with the Nanoscan in the two upper channels over 200 nm are potentially misclassified in the radial differential mobility analyzer (37). Therefore, the two upper channels of the NanoScan size distributions should be interpreted carefully (e.g., Figures 4C,D).

### Comparison of Worker Exposure Concentrations With Recommended Exposure Limits

Table 2 represents the background-corrected 8h-TWA worker exposure to total particle number and respirable dust concentrations calculated by Equation (1) during a typical working day. The calculated 8h-TWA exposure, in terms of mass concentration, was 0.02 mg m^−3^ (Table 5). For the 8h-TWA exposure calculation, it was assumed that the daily equivalent working time during post-processing of a 3D printed metal object comprised of 2 h in the NF, and the rest of working hours spent in FF.

**Table 5:** Background corrected 8 h-TWA respirable dust (RD) exposure concentrations obtained for a typical working day, and comparison with the permissible exposure limit (PEL) for respirable inert mineral dust per The Danish Working Environment Authority (40).

	**RD_**8 h-TWA**_ [mg m^**−3**^]**	**PEL_**8 h-TWA**_ [mg m^**−3**^] (40)**	**RD_**8 h-TWA**_/PEL_**8 h-TWA**_**
Working day at the facility	0.02	5	0.004

The Danish Working Environment Authority (40) set a permissible exposure limit (PEL) for 8h TWA of 10 mg m^−3^ and of 5 mg m^−3^ for total and respirable inert mineral dust, respectively. For TiO_2_ the current Danish total suspended dust limit is 10 mg m^−3^. The National Institute for Occupational Safety and Health (NIOSH) has proposed a recommended exposure limit (REL) of 2.4 mg m^−3^ for fine TiO_2_ (defined as <2.5 μm diameter) in workplace air on the basis of available toxicity data (41). The measured exposure levels is also below the 0.3 mg/m^−3^ suggested by NIOSH researchers for ultrafine TiO_2_ (42). Even though the tested feedstock material has a moderate respirable dust release fraction of 171 ± 5.1 mg kg^−1^, in our measurements, the mass exposure level of respirable particles was well below both the permissible and recently suggested exposure limit for fine and ultrafine TiO_2_.

## Conclusions

In the present work, particle release from activities related to 3D metal printing was investigated. The activities included emptying and cleaning of the printing chamber after printing, removal of excess powder material in an either open or closed system, baking for annealing the metal object, and grinding and preparation of the printing substrate for the base of the next printed object. Two of the five activities i.e., the closed powder removal and initial annealing process did not cause particle number concentrations to increase significantly over measured background particle concentrations within the measured particle range. Concentrations when emptying the chamber and during the open PRS cleaning processes increased to 10^4^ and 5·10^3^ cm^−3^, respectively, whereas grinding activity increased number concentrations up to 2.5·10^5^ cm^−3^ as measured by the CPC in the NF. In the FF, particle concentrations reached 1.5·10^3^, 2.5·10^3^, and 1.5·10^4^ cm^−3^ for the chamber emptying open PRS cleaning processes, and the substrate grinding activity, respectively. Size distributions showed that particles released as a result of the activities were mainly below 200 nm in diameter, with average particle size in the BZ being between 53 and 70 nm as measured by the DiscMini. Grinding of the base printing substrate caused a significant release of particles with a mode at 50–80 nm. Particles with diameters larger than 500 nm released during the grinding process were attributed to droplets of the cooling solution used during the activity. Respirable mass concentrations collected on filters were 50.4 μg m^−3^. This was corroborated by respirable mass measured with a DustTrak of 58.4 μg m^−3^. Respirable 8 h time-weighted average mass concentrations were calculated to 0.02 mg m^−3^, which were 0.4% of the occupational exposure limits of 5 mg m^−3^ for respirable mineral dust in Denmark for a workday, but 6.67% of the REL for ultrafine TiO_2_.

While this study only covers a single case study of metal 3D printing method, it covers several pre- and post-processes included as part of the 3D printing process. Thereby, this study provides valuable insight into the particle release during processes related to metal 3D printing and aids to improve understanding of the potential exposure in working environments where metal 3D printing occurs.

## Data Availability Statement

The raw data supporting the conclusions of this article will be made available by the authors, without undue reservation.

## Author Contributions

AJ did data analysis and drafted the manuscript. HH planned and performed measurements. AB conducted EM work and wrote the relevant sections for the manuscript. KJ planned and created the financial support to conduct the measurements. AF planned and performed measurements and did exposure calculations. All authors commented and corrected the manuscript and accepted the final version.

## Conflict of Interest

HH was employed by company Joblife A/S. The remaining authors declare that the research was conducted in the absence of any commercial or financial relationships that could be construed as a potential conflict of interest.
