# Supplementary material for: Nanoparticle Exposure and Workplace Measurements During Processes Related to 3D Printing of a Metal Object
Source: Front Public Health. 2020 Nov 25;8:608718. doi: 10.3389/fpubh.2020.608718 (PMC7723871; doi:10.3389/fpubh.2020.608718)
Supplement: Supplementary file 1 [file Table_1.DOCX]

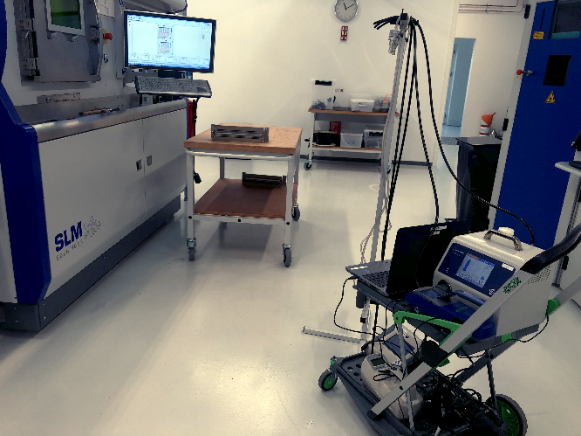


**A**


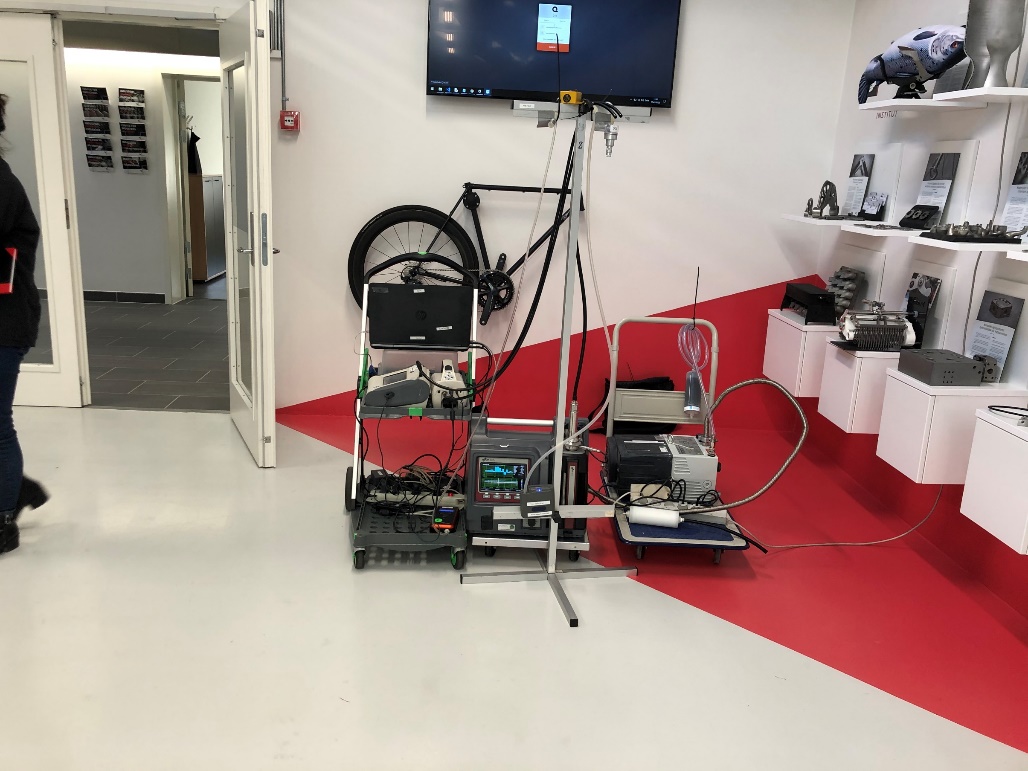


**B**


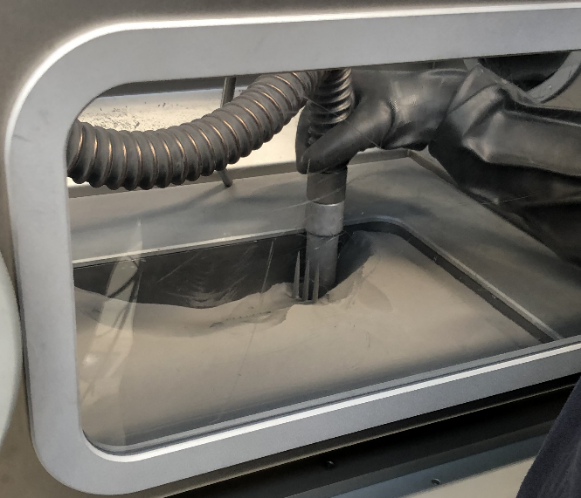


**C**


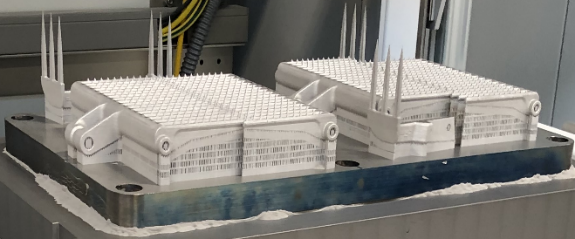


**D**


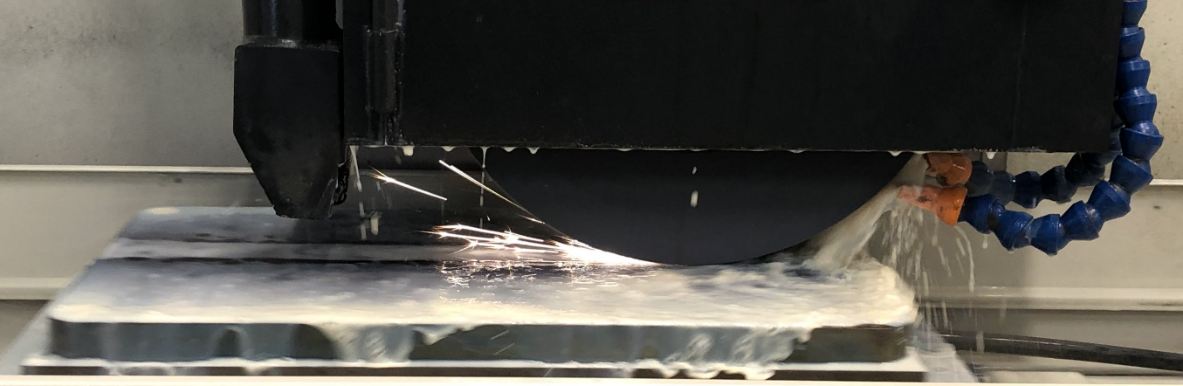


**E**

*Figure S1. SLM 500 3D printing machine with movable NF measurement position (A); Static Far field measurement position (B); Powder removal system (PRS) in closed state (C); Printed 3D object (D); Spark generation during the grinding of the base plate (E).*
